# Chemical Modification of B_4_C Films and B_4_C/Pd Layers Stored in Different Environments

**DOI:** 10.3390/ma14051319

**Published:** 2021-03-09

**Authors:** Yufei Feng, Runze Qi, Li Jiang, Qiushi Huang, Tongzhou Li, Genchang Liu, Wenbin Li, Wensheng Yan, Zhong Zhang, Zhanshan Wang

**Affiliations:** 1 MOE Key Laboratory of Advanced Micro-Structured Materials, Institute of Precision Optical Engineering (IPOE), School of Physics Science and Engineering, Tongji University, Shanghai 200092, China; 1810089@tongji.edu.cn (Y.F.); 1986jiangli@tongji.edu.cn (L.J.); huangqs@tongji.edu.cn (Q.H.); litongzhou@tongji.edu.cn (T.L.); liugenchang@tongji.edu.cn (G.L.); wbli@tongji.edu.cn (W.L.); zhangzhongcc@tongji.edu.cn (Z.Z.); wangzs@tongji.edu.cn (Z.W.); 2 National Synchrotron Radiation Laboratory, University of Science and Technology of China, Hefei 230029, China; ywsh2000@ustc.edu.cn

**Keywords:** B_4_C film, B_4_C/Pd layers, catalysis, storage environment, XANES

## Abstract

B_4_C/Pd multilayers with small d-spacing can easily degrade in the air, and the exact degradation process is not clear. In this work, we studied the chemical modification of B_4_C films and B_4_C/Pd double layers stored in four different environments: a dry nitrogen environment, the atmosphere, a dry oxygen-rich environment, and a wet nitrogen environment. The XANES spectra of the B_4_C/Pd layers placed in a dry oxygen-rich environment showed the most significant decrease in the σ* states of the B–C bonds and an increase in the π* states of the B–O bonds compared with the other samples. X-ray photoelectron spectroscopy (XPS) measurements of the samples placed in a dry oxygen-rich environment showed more intensive B-O binding signals in the B_4_C/Pd layers than in the single B_4_C film. The results of the Fourier-transform infrared spectroscopy (FTIR) showed a similar decrease in the B–C bonds and an increase in the B–O bonds in the B_4_C/Pd layers in contrast to the single B_4_C film placed in a dry oxygen-rich environment. We concluded that the combination of palladium catalysis and the high content of oxygen in the environment promoted the oxidization of boron, deteriorated the B_4_C composition.

## 1. Introduction

The photon flux of multilayer monochromators on synchrotron beamlines is approximately two orders of magnitude larger than that of crystal monochromators [1,2]. The most distinct advantage of these techniques is that they do not require high energy resolution, such as X-ray fluorescence spectroscopy (XRF) [3,4], small-angle X-ray scattering (SAXS) [5,6], and X-ray micro-imaging [7,8]. There are many typical multilayer material combinations for monochromators, for example, Si/W [9,10], B_4_C/W [4,11,12], and B_4_C/Pd [8,13,14]. B4C/Pd multilayers, which have high reflectivity and good energy resolution at approximately 7–15 keV, have been used in synchrotron beamlines, such as TopoTomo (ANKA light source) [14]. Recently, Ni et al. have fabricated the ultrathin B_4_C/Pd multilayers with d-spacing of 2.5 nm by magnetron sputtering using the heavy noble gas Kr to improve the layer structure [15]. The excellent performance of B_4_C/Pd multilayers are suitable to be used as a monochromator.

However, small d-spacing B_4_C/Pd multilayers are unstable under an atmospheric environment. Morawe et al. reported that B_4_C/Pd multilayers with d-spacing of approximately 3 nm degraded in the air after only a few days without protective layers [16,17]. The first peak of measured X-ray reflectivity curve broadened and shoulders appeared at higher angles indicating the compaction of the B_4_C layers and destruction of the periodic structure. With larger d-spacing, multilayers deposited by reactive sputtering using an Ar and N_2_ gas mixture were also unstable compared to other nitridated multilayers, like B_4_C+N/Ru [18,19] and B_4_C+N/W multilayers [20]. Wang et al. reported that nitridated B_4_C/Pd multilayers with d-spacing of approximately 5 nm decayed after the samples were stored in an air environment for 6–17 months [21].

We found that many layers near the surface deteriorated with severe interdiffusion and compaction of the layers. This is a significant issue for applications, as most multilayers for hard X-ray monochromator have d-spacing less than 3 nm, and the conditions for preparation, storage, and installation become critical. Although certain protective layers have been developed to elongate the lifetime of B_4_C/Pd multilayers [16], there is no further investigation of the effects of different environmental factors, the contribution from different materials, and the composition changes of films in the degradation process, which is essential for the future development and applications of B_4_C/Pd multilayer monochromators.

The oxidation of B_4_C as an important subject has been studied in many literatures. Zehringer et al. [22] reported that exposure of sputter-cleaned surfaces of boron-carbide to molecular oxygen at room temperature did not cause a measurable surface oxidation. Li et al. [23] found Gibbs energy of the reaction between B_4_C and oxygen was great than that of the reaction between B_4_C and water vapor at the same temperature. Viricelle et al. [24] reported that the oxidation rate of B_4_C increases and is enhanced by water vapor at 700 and 800 °C. To sum up, B_4_C is very stable at room temperature, and will oxidize with oxygen and water at a high temperature.

Palladium is typically used as a catalyst in chemistry. Nicolaou et al. reported that palladium catalyzed carbon–carbon bond formation in most common synthesis reactions [25]. Tat Thang Vo Doan et al. [26] and Li et al. [27] found that B–Pd nanocrystals reduced O=O bond reaction barriers and strengthened oxygen reduction reactions. Palladium catalysis promotes the dissociation of oxygen and reduces the oxidation reaction potential. Therefore, we can assume that the presence of Pd promotes B_4_C oxidation.

In this paper, we first confirm the hypothesis of the catalytic effect of Pd on B_4_C/Pd layered systems and further explore the degradation process by investigating the stability of B_4_C films and B_4_C/Pd double layers stored in different environments. We fabricated two groups of samples of a single B_4_C film and a B_4_C layer with a Pd layer underneath. Both groups of samples were placed in different environments for 50 days. The samples were then measured using X-ray absorption near-edge structure (XANES) spectroscopy, X-ray photoelectron spectroscopy (XPS), and Fourier-transform infrared spectroscopy (FTIR) to analyze the changes in the chemical state after film degradation. The results indicated that boron combined with oxygen and boron carbide degraded under the action of palladium in the oxygen environment. The work is a useful guide for exploring the oxidation process of B_4_C materials with nano-film state, and for the protection and application of ultrathin B_4_C/Pd multilayers in synchrotron monochromators.

## 2. Experimental Techniques

### 2.1. Sample Preparation

The direct current (DC) magnetron sputtering technique was developed to prepare samples. Each magnetron cathode was operated at 100 W and 15 W for the B_4_C and Pd targets, respectively. The samples were deposited at Ar gas pressures of 1 mTorr at room temperature and the background pressure was lower than 5.5 × 10^−5^ Pa to avoid the incorporation of oxygen into the sputtering process. Firstly, the deposition rate of B_4_C and Pd was calibrated by measuring and fitting the X-ray reflectivity curve of single B_4_C and Pd films. Known from our previous investigation [28], the stoichiometric ratio of B:C was around 4:1 as the B_4_C layer sputtered at Ar gas pressures of 1 mTorr. The thin B_4_C film obtained under this sputtering condition is very dense, with a density (2.39 g/cm^3^) equivalent to 95% of the bulk B_4_C material (2.52 g/cm^3^). Then two groups of samples were fabricated. In the first group of samples, a 10 nm B_4_C film was deposited directly on a Si substrate. In the second group, a 1 nm Pd layer was added between a 10 nm B_4_C layer and a Si substrate to prove the function of Pd. After preparation, the samples were immediately transferred to the corresponding storage environment.

### 2.2. Storage Environment Conditions

To explore the factors that degrade films, the B_4_C films and B_4_C/Pd layers were sealed in boxes and stored in four different environments for 50 days: (A) A dry nitrogen environment (pumped down and filled with nitrogen), which is isolated from O_2_ and H_2_O and can be used as a reference. (B) The atmosphere, which is the assumed working environment of the optics. (C) A dry oxygen-rich environment (pumped down and filled with oxygen), where oxygen is the impact factor. (D) A wet nitrogen environment (pumped down, humidified, and filled with nitrogen), where H_2_O is the impact factor. Detailed parameters of the storage environments are listed in Table 1. The samples stored in a dry nitrogen environment, isolated from O_2_ and H_2_O, can be used as a reference.

### 2.3. The X-ray Absorption Near-Edge Structure (XANES)

X-ray absorption near-edge structure (XANES) spectroscopy is commonly used to probe the local atomic structure around specific elements in films and often used as a fingerprint of the changes in the coordination numbers of elements [29,30,31,32,33]. XANES measurements were conducted 50 days after fabrication at the Beamline U12b at the National Synchrotron Radiation Laboratory (NSRL) in the total electron yield (TEY) mode by collecting sample drain currents under a vacuum at greater than 5 × 10^−8^ Pa. The beam from a bending magnet was monochromatized with a varied line-spacing plane grating and refocused using a toroidal mirror. An energy range from 100 to 1000 eV was used with an energy resolution of approximately 0.2 eV [34].

All XANES spectra were measured using the process of background subtraction and normalization by considering the low and high photon energy parts of the spectra far from the threshold [32].

### 2.4. X-ray Photoelectron Spectroscopy (XPS)

To study the changes in the electronic structures of the films’ elements after degradation, the XPS measurements were conducted using a Thermo Fisher Scientific K-Alpha+ spectrometer (Thermo Fisher Scientific, Waltham, MA, USA) with an Al-Kα characteristic emission line (photon energy E = 1486 eV) [35]. The B 1s, C 1s, and O 1s core-level spectra were investigated after the single B_4_C films and B_4_C/Pd layers were placed in the dry oxygen-rich environment. The Pd 3d core-level spectra at the interface of the Pd and B_4_C layers were also investigated to explore the chemical state of Pd.

### 2.5. Fourier-Transform Infrared Spectroscopy (FTIR)

Fourier-transform infrared spectroscopy was recorded on a Fourier-transform infrared spectrometer (FTIR, Tensor 27). First, a spectrum of the Si substrate was collected as the background spectrum. The spectra of the single B_4_C films and B_4_C/Pd layers placed in the dry oxygen-rich environment were then obtained under the same test conditions. The data processing consisted of two steps: Background subtraction and position correction based on the peaks of the Si–Si bonds [36].

## 3. Experimental Results

### 3.1. XANES Measurements

#### 3.1.1. B K-Edge XANES

**Figure 1:**
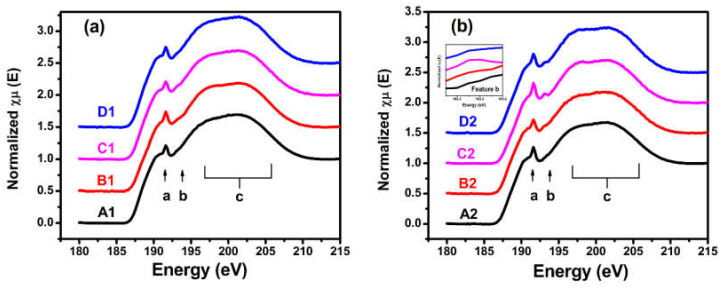
The B K-edge X-ray absorption near-edge structure (XANES) spectra of the B_4_C films (**a**) and B_4_C/Pd layers (**b**) in the different storage environments. Samples A1 and A2 were placed in a dry nitrogen environment; samples B1 and B2 were placed in the atmosphere; samples C1 and C2 were placed in a dry oxygen-rich environment; and samples D1 and D2 were placed in a wet nitrogen environment. The insert in Figure 1b is an enlarged view of feature b in the B_4_C/Pd layers.

The B K-edge X-ray absorption near-edge structure spectra of the samples placed in the different storage environments are shown in Figure 1. The spectra had three features: One peak at 191.6 eV, one minor sharp oscillation at 193 eV, and one broad slope from 196.0 eV to 208.0 eV that were marked as a, b, and c, respectively. The spectral features at the different energy positions represented different chemical states and the local atomic structure of boron. Peak a at 191.6 eV was due to the transition of B 1s electrons to unoccupied B 2p states of B_4_C [37]. Feature b at 193 eV was attributed to the localized π* states of B_2_O_3_ [37,38,39], and the broad feature c from 196.0 eV to 208.0 eV was due to the σ* states of the B-C bonds [38,39,40,41]. As shown in Figure 1a, the three spectral features of the B1, C1, and D1 samples of the single B_4_C films underwent no significant changes compared with the A1 sample placed in the dry nitrogen environment.

The results indicate that the local atomic structure around the boron experienced almost no changes in the single B_4_C films even in the different environments. Figure 1b shows that the three spectral features of the B_4_C/Pd layers placed in the dry oxygen-rich environment (C2 sample) changed significantly compared with the A2 sample placed in the dry nitrogen environment. Peak a at 191.6 eV in the C2 sample’s spectrum was more intense and sharper than the other samples, indicating that some boron atoms became unstable in the B_4_C/Pd layers placed in the dry oxygen-rich environment.

The peak position of feature b was related to the π* states of the B_2_O_3_, suggesting that some of the boron combined with oxygen. This change agreed with the results of the O K-edge XANES presented in Figure 4, with peaks at 536.1 eV [39,42,43]. The decline in feature c, assigned to the σ* states of the B–C bonds, indicated that some B–C chains were breaking in the film [38,39]. The corresponding change also appeared in the C K-edge XANES signal with a peak at 284.5 eV and a slope from 291 to 298 eV [40,41] as shown in Figure 3. These spectral features demonstrated that, in the B_4_C/Pd layers placed in the oxygen-rich environment, the local atomic structure and chemical states of the boron changed.

The spectral features of the B_4_C/Pd layers placed in the atmosphere and wet nitrogen environment (the B2 and D2 samples, respectively) changed little compared with the B_4_C/Pd layers placed in the dry nitrogen environment (A2 sample). There was almost no increase in the B2 sample’s spectrum due to the low concentration of oxygen in the atmosphere. As no obvious changes were observed in the D2 sample’s spectrum, H_2_O appeared to have less effect on the degradation of the layers compared to O_2_.

**Figure 2:**
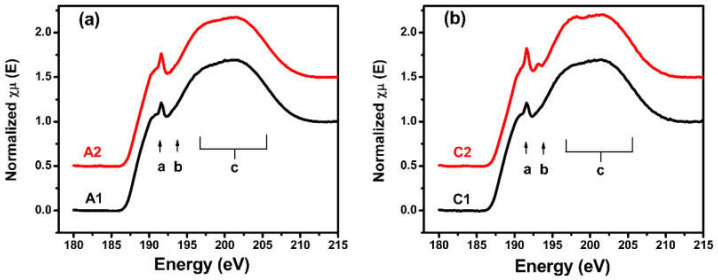
The B K-edge XANES spectra of the B_4_C films (A1 and C1) and B_4_C/Pd layers (A2 and C2) placed in the dry nitrogen environment (**a**) and in the dry oxygen-rich environment (**b**).

To further prove the effects of palladium and oxygen on the degradation of the films, the spectra of different films in the same storage environments are demonstrated in Figure 2. Figure 2a shows the spectra of the single B_4_C film and B_4_C/Pd layers placed in the dry nitrogen environment. There was no obvious difference in the spectral features between the two samples, proving that the B_4_C/Pd layers were stable without the action of oxygen. Figure 2b shows the spectra of the single B_4_C film and the B_4_C/Pd layers placed in the dry oxygen-rich environment. The spectral features, as previously mentioned, of the B_4_C/Pd layers showed obvious changes, indicating that the presence of palladium degraded the film in the dry oxygen-rich environment.

#### 3.1.2. C K-Edge XANES

**Figure 3:**
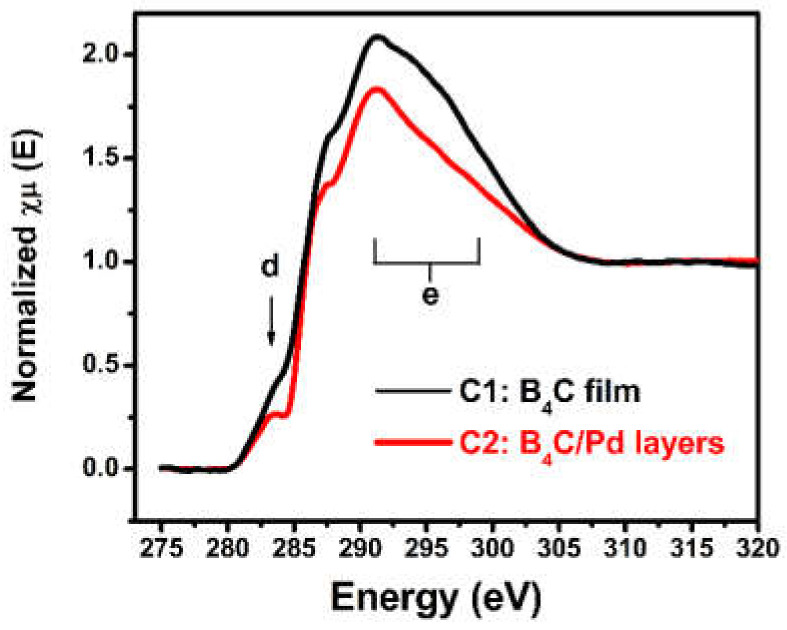
The C K-edge XANES spectra of the B_4_C film and B_4_C/Pd layers placed in the dry oxygen-rich environment.

The C K-edge X-ray absorption near-edge structure spectra were measured to analyze the change of local atomic structure around the carbon element. Figure 3 shows the C K-edge X-ray absorption near-edge structure spectra of the different films placed in the dry oxygen-rich environment. The C 1s absorption edge of the B_4_C film was characterized by the antibonding π* state of the C–B bonds at 284.5 eV and the σ* states of the C–B bonds from 291 to 298 eV, symbolized by d and e, respectively [40,41]. The most significant change in the C 1s spectrum of the B_4_C/Pd layers was the decrease in the intensity of the antibonding π* state and the σ* state compared with the spectrum of the B_4_C film. The decrease proved that the part of C–B chain was breaking due to the presence of Pd in the oxygen-rich environment and as a result, part of the B_4_C composition was destroyed.

#### 3.1.3. O K-Edge XANES

**Figure 4:**
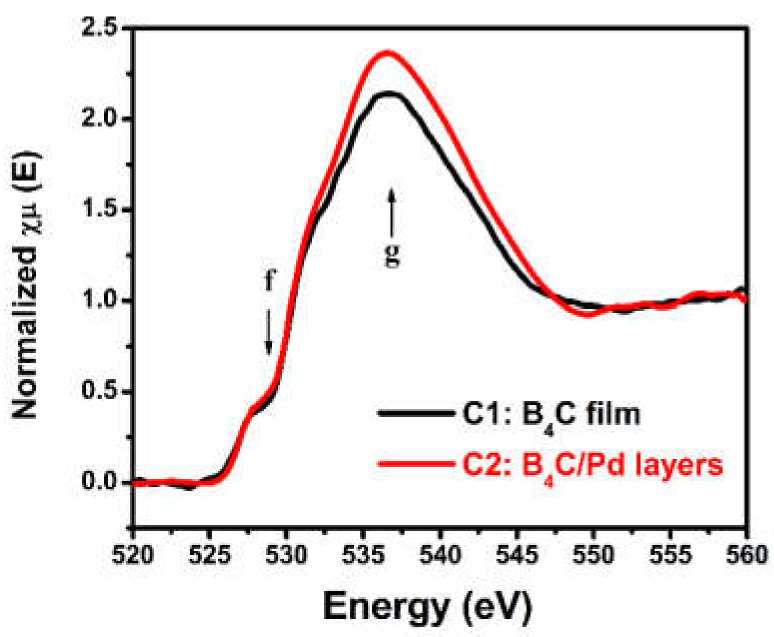
The O K-edge XANES spectra of the B_4_C film and B_4_C/Pd layers placed in the dry oxygen-rich environment.

To prove the effect of oxygen on the film, the O K-edge X-ray absorption near-edge structure spectra were also measured. Figure 4 displays the O K-edge X-ray absorption near-edge structure spectra of the different films placed in the dry oxygen-rich environment. The spectra had two significant features: one small bump due to the π* state of the O_2_ molecule at 529.5 eV [42,43,44,45], labeled f, and another broad feature attributed to the σ* state of the B–O bonds at 536.1 eV [43,44,45], labeled g. There was a significant increase in the σ* states of the B–O bonds in the spectrum of the B_4_C/Pd layers compared with the spectrum of the B_4_C film, which indicated that some of the oxygen was excited and combined with boron under palladium catalysis. The same O_2_ peak occurred in the spectra because the samples were placed in a dry oxygen-rich environment.

Summarizing the XANES results, there was a reasonable explanation of the degradation of the B_4_C/Pd layers: Under palladium catalysis, some of oxygen was excited [27], replacing some of the carbon atoms around the boron and combining with the boron, which caused the deterioration of the B_4_C composition.

### 3.2. XPS Measurements

**Figure 5:**
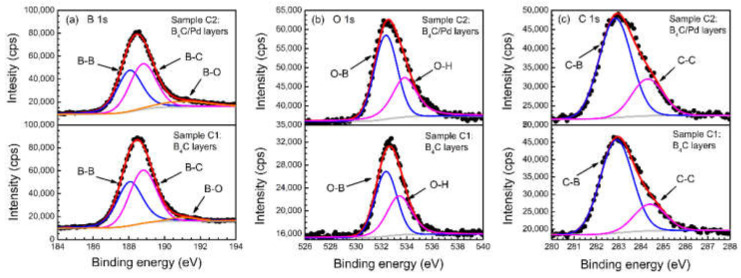
The X-ray photoelectron spectroscopy (XPS) spectra of the B_4_C film and B_4_C/Pd layers placed in the dry oxygen-rich environment. (**a**) B 1s, (**b**) O 1s, and (**c**) C 1s.

Examinations of the XPS spectra around the B 1s, O 1s, C 1s, and Pd 3d regions provided more details about the electronic structures of the film elements. Figure 5 presents the B 1s, O 1s, and C 1s spectra of the single B_4_C film and B_4_C/Pd layers placed in the dry oxygen-rich environment. Both test positions were approximately 5 nm from the surface of the B_4_C layer to avoid surface contamination. All photoelectron peaks were fitted by a weighted least-squares fitting method using Lorentzian–Gaussian line shapes after background subtraction according to the Shirley method. The peak width of the fitted spectra of B_4_C film was consistent with the B_4_C/Pd layers.

Figure 5a presents the B 1s spectra of the single B_4_C film and B_4_C/Pd layers. In the spectrum of the B_4_C/Pd layers, there was a weak slope at a binding energy of 192.5 eV due to the B–O component [27,46,47], which indicated that some of the boron (~9% in atomic fraction) combined with oxygen under the action of palladium. A small B–O component (~2% in atomic fraction) was also found by fitting the B 1s spectra of the single B_4_C film. The B 1s spectra also show the reduction of B–B bonds (from about 48% down to 45% in the atomic fraction) and B–C bonds (from about 50% down to 46% in the atomic fraction) at binding energies of 188 and 189 eV, respectively [46,47,48].

Figure 5b shows the O 1s spectra of the single B_4_C film and B_4_C/Pd layers. The signal peak was due to the O–B bonds at 531.7 eV [49] and the O–H bonds at 533.2 eV [50] from the adsorbed water molecules. The amount of O–B bonds in the spectrum of the B_4_C/Pd layers (~58% in the atomic fraction) were higher than in the spectrum of the single B_4_C film (~54% in the atomic fraction). The results indicate that a larger ratio of oxygen reacted with boron under the presence of palladium, which is consistent with the results of the B 1s spectra. The stoichiometric ratio of O:B was around 1:9.8 in the B_4_C/Pd layers, which was larger than in the single B_4_C films (1:18.8). This shows that the content of oxygen in the B_4_C/Pd layers significantly increased under the catalysis of palladium. The results also indicate that the amount of oxidized boron in the B_4_C/Pd layers increased significantly.

Figure 5c demonstrates the XPS spectra of C 1s in the single B_4_C film and B_4_C/Pd layers. The C 1s spectra had peaks at binding energies of 283.0 eV and 284.5 eV due to the C–B bonds and C–C bonds, respectively [46,47,48]. There was a reduction in the C–B bonds in the spectrum of the B_4_C/Pd layers (~66% in atomic fraction) compared with those in the spectrum of the B_4_C film (~71% in atomic fraction), indicating that some of the C–B bonds broke due to the reaction of B with O in the B_4_C/Pd layers. This result was consistent with the C K-edge XANES analysis.

**Figure 6:**
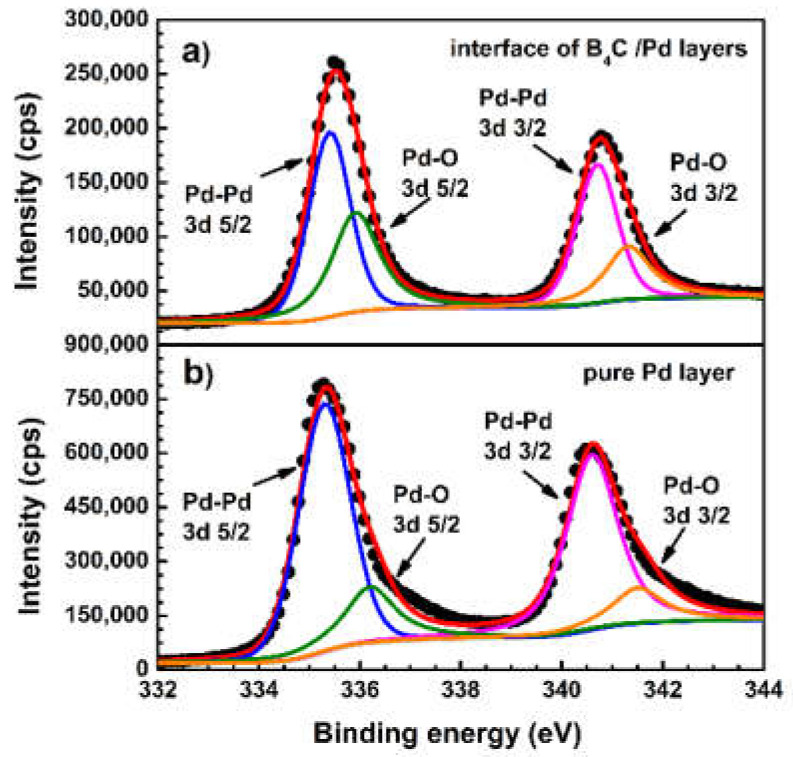
The XPS spectra of the Pd 3d: (**a**) at the interface of the Pd layer and B_4_C layer placed in the dry oxygen-rich environment; and (**b**) pure Pd layer.

To further explore the changes of palladium, the XPS spectra of Pd 3d were also measured. Figure 6a shows the XPS spectrum of the Pd 3d at the interface of the B_4_C/Pd layers placed in the dry oxygen-rich environment. The spectrum of the Pd 3d at the position approximately 5 nm from the surface of single Pd layer, as reference, is also shown in Figure 6b. All photoelectron peaks were fitted by a weighted least-squares fitting method using Lorentzian–Gaussian line shapes after background subtraction according to the Shirley method. In the spectrum of the pure Pd layer, the peak was fitted as the 3d 5/2 and 3d 3/2 levels of the Pd-Pd bonds located at 335.3 eV and 340.6 eV [27,51,52], and a small portion of the 3d 5/2 and 3d 3/2 levels of the Pd–O bonds located at 335.9 eV and 341.3 eV [27,51,52]. The atomic fraction of the Pd–O bonds was about 21%.

For the spectrum of the Pd 3d at the interface of the B_4_C/Pd layers placed in the dry oxygen-rich environment, the peaks were slightly shifted to a higher energy position due to the enhancement of the Pd–O bonds signal. The atomic fraction of the Pd–O bonds was about 39%. The increase of the Pd–O bonds indicates that more Pd atoms were excited. Therefore, Pd will be more involved in the reaction in the B_4_C/Pd layers placed in the dry oxygen-rich environment. The same result also appeared in other Pd catalytic experiments [26,27], demonstrating that, when Pd, B, and O coexist, Pd donates electrons to O and B transfers electrons to O, indicating that Pd and B are involved in the decomposition of O and promotes oxygen reduction reactions.

### 3.3. FTIR Absorption Spectra

**Figure 7:**
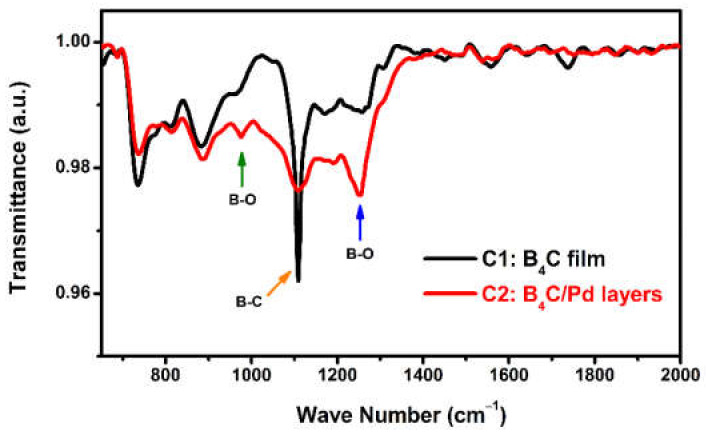
The Fourier-transform infrared spectroscopy (FTIR) absorption spectra of the B_4_C film and B_4_C/Pd layers placed in the dry oxygen-rich environment.

To further investigate the vibration of bonds, the FTIR absorption spectra were recorded. Figure 7 shows the FTIR absorption spectra of the single B_4_C film and B_4_C/Pd layers placed in the dry oxygen-rich environment. The spectrum of the B_4_C film had a strong absorption peak near 1100 cm^−1^ that was due to the B–C bonds in the B_4_C composition [53,54,55,56]. In the spectrum of the B_4_C/Pd layers, the peak of the B–C bonds decreased significantly, indicating that some of B–C chains were breaking due to the presence of the Pd in the oxygen-rich environment. In addition, there were two new peaks in the spectrum of the B_4_C/Pd layers, minor features in the regions of 950–1000 cm^−1^, and absorption bands in the regions of 1200–1300 cm^−1^ due to the B–O modes [54,55,56], indicating that some of the boron was oxidized. The FTIR absorption spectra confirmed that part of B_4_C composition was destroyed and that boron combined with oxygen in the dry oxygen-rich environment under palladium catalysis.

## 4. Discussion

The three measurements showed the same results indicating that the B_4_C composition tended to degrade under palladium catalysis in the oxygen environment. Considering that the film is only a few nanometers, these characterizations are enough to clearly prove the effect of Pd and O on this degradation process. A simple degradation process could be assumed as follows:

1. First oxygen diffused into the film. Known from the previous calculation of density functional theory (DFT), O_2_ dissociation proceeds on a Pd layer with reaction barriers of 0.72 eV [26]. The energy of the reaction barriers decreases to 0.63 eV when B and Pd exist simultaneously [26], indicating B and Pd enhance the decomposition of O and promote the oxidation reaction. In our work, the most obvious structural change occurs in the B_4_C/Pd layers placed in a dry oxygen-rich environment. In this case, it is essential that numerous O_2_ will decompose into O with the participation of B and Pd.
2. Then, dissociated oxygen replaced the carbon around boron and combined with boron. The formation of B_2_O_3_ (Δ*_f_H*° = −1194 kJ/mol) releases a larger amount of energy than B_4_C (Δ*_f_H*° = −71 kJ/mol) [16], indicating that boron prefers to combine with oxygen. In other B_4_C oxidation experiments [23], at elevated temperatures, carbon atoms will form carbon dioxide. While in our experiment, at room temperature, carbon atoms can only be in a non-excited state [17]. Thus, we could assume that the reaction product prefers boron oxide and carbon.
3. Finally, if water vapor exists, B_2_O_3_ will react to form H_3_BO_3_, and then volatilize [24], leading to the reduction in the B content in the film.

As a result, the B_4_C layer is deteriorated and degraded. As for the B_4_C/Pd multilayers, the reduction of B atoms leads to the destruction of the periodic structure and the degradation of the optical performance [16,17].

More theoretical and experimental research are required to explore the evolution of the chemical modifications in films. The sample in this study was only a double-layer structure with a thick B_4_C top layer. These structures can be more stable compared with nanoscale multilayer structures in which each layer is only ~1 nm thick [16]. In future, we will investigate the effect of environmental factors, including oxygen and H_2_O, on real B_4_C/Pd multilayers.

## 5. Conclusions

In summary, we explored the stability of B_4_C films and B_4_C/Pd layers placed in a dry nitrogen environment, the atmosphere, a dry oxygen-rich environment, and a wet nitrogen environment. The chemical state of the films was analyzed using XANES, XPS, and FTIR. There was no significant change in the XANES spectra of the single B_4_C films, suggesting that the single B_4_C film was stable even under different environments. In the sample with B_4_C/Pd layers placed in the dry oxygen-rich environment, the XANES spectra demonstrated a significant increase in the π* states of the B–O bonds and a decrease in the σ* states in the B–C bonds, indicating that the boron was oxidized and that the B_4_C composition was destroyed under the action of both palladium and oxygen. The XPS results also proved that the amount of oxidized boron in the B_4_C/Pd layers increased due to Pd catalysis. The FTIR results provided the same conclusions as XANES and XPS. These analyses reasonably explain the degradation of the B_4_C/Pd layers in a high-oxygen environment, which should be avoided during storage. This study provides useful guidance for the further development and applications of B_4_C/Pd multilayers.

## Figures and Tables

**Table 1:** Detailed parameters of the storage environments.

Environments	Temperature/°C	Humidity/%rh
A: Dry nitrogen environment	20 °C	~25%rh
B: Atmosphere	20 °C	~35%rh
C: Dry oxygen-rich environment	20 °C	~25%rh
D: Wet nitrogen environment	20 °C	~85%rh

## Data Availability

No new data were created or analyzed in this study. Data sharing is not applicable to this article.
